# Incidence of Microscopically Positive Proximal Margins in Adenocarcinoma of the Gastroesophageal Junction

**DOI:** 10.1371/journal.pone.0088010

**Published:** 2014-02-05

**Authors:** Fei Gao, Jia Chen, Tao Wang, Gang Wang, Zhihong Zhang, Lizong Shen

**Affiliations:** 1 Division of Gastrointestinal Surgery, Department of General Surgery, First Affiliated Hospital, Nanjing Medical University, Nanjing, Jiangsu, China; 2 Department of Pathology, First Affiliated Hospital, Nanjing Medical University, Nanjing, Jiangsu, China; University of Torino, Italy

## Abstract

**Aim:**

To investigate the incidence and risk factors of microscopically positive proximal margins in Chinese patients with adenocarcinoma of the gastroesophageal junction.

**Methods:**

The medical records of 483 patients, who underwent surgical treatment with curative intent for adenocarcinoma of the gastroesophageal junction in a single high-volume tertiary medical center, were reviewed. Demographic, radiographic, endoscopic, pathologic, and treatment-related variables were evaluated. All proximal margins were re-evaluated by two experienced pathologists, and a positive proximal margin was defined as the microscopic presence of invasive tumor cells seen at the esophageal transaction margin submitted *en face* on final paraffin sections.

**Results:**

The incidence of positive proximal margins was 23.81% in this series. Siewert type, depth of tumor invasion, lymph node involvement, presence of vascular or lymphatic invasion, and presence of perineural invasion were significantly associated with positive proximal margins. On multivariate analysis, the presence of vascular or lymphatic invasion and advanced-stage disease were independent risk factors for positive proximal margins in patients with adenocarcinoma of the gastroesophageal junction.

**Conclusion:**

Residual cancer at proximal resection margins remains a major issue for the surgical treatment of adenocarcinoma of the gastroesophageal junction in China.

## Introduction

Gastric cancer remains one of the most prevalent causes of cancer-related death in China [1]. Adenocarcinoma of the gastroesophageal junction (AGE) is a significant clinical problem and has been regarded as a separate entity because it appears to have distinct prognostic and pathological features [2]. The prevalence of AGE continues to dramatically increase in Western populations [2]–[6].

Siewert and Stein [7] proposed a widely approved classification of AGE in 1996 that divided AGE into three subgroups based on the distance between the tumor epicenter and the esophagogastric junction line (EGJ). Type I is defined as a tumor in which the center is located 1 to 5 cm above the EGJ, regardless of invasion to the EGJ. Type II tumors straddle the EGJ line and are believed to be true EGJ cancers in which the epicenter is located between 1 cm above and 2 cm below the EGJ. Finally, type III tumors are subcardial gastric cancers invading the EGJ, with the epicenter 2 to 5 cm below the EGJ.

Surgical extirpation of the primary tumor remains the standard curative treatment of patients with AGE. However, tumor infiltration of the proximal resection margin has been associated with diminished survival in most series [4]–[6], [8], [9]. Of all prognostic factors associated with overall survival of patients with AGE, the proximal resection margin status is the variable most consistently reported [5]. Importantly, the margin status is also the variable that is most likely to be impacted by surgical technique. Achieving a clear proximal margin should be the goal of surgical therapy for AGE.

Although many studies on the proximal margin for AGE have been performed, there are few studies concerning the proximal margin status of patients with AGE in China. The aim of the present retrospective study was to investigate the incidence and risk factors of positive proximal margins in a single high-volume tertiary medical center in Nanjing, China. It is anticipated that this study will provide a rational basis for the most appropriate surgical approach for curative treatment of AGE in China.

## Materials and Methods

This retrospective study was approved by the Nanjing Medical University Institutional Review Board. Written consent was given by the patients for their information and samples to be stored in the hospital database and used for research. This study was also in compliance with the Helsinki Declaration.

A total of 494 consecutive patients with AGE who underwent resection from January 2010 through December 2011 were identified through a search of the computerized pathology database established in the Department of Pathology in the First Affiliated Hospital of Nanjing Medical University, Nanjing, China. AGE was defined as an adenocarcinoma with its center within 5 cm proximal and distal to the anatomic gastroesophageal junction (GEJ) and was divided into three types according to Siewert’s classification. No patients underwent neoadjuvant chemotherapy or radiotherapy. Seven patients who underwent palliative resection, and four with both AGE and esophageal cancer were excluded from the analysis. Therefore, 483 patients who underwent surgical treatment with curative intent for AGE were enrolled in this study. The curative intent means that the surgical approaches are performed with the goal of achieving complete macroscopic and microscopic tumor resection. The medical records of these patients, including demographic, radiographic, endoscopic, pathologic, and treatment-related variables, were reviewed. Tumor location was determined by esophagoduodenogastroscopy, upper gastrointestinal barium examination, abdominal computed tomography, the surgical presentation, and the pathologic report.

### Surgical Approach

All patients underwent tumor resection at the Department of Surgery in the First Affiliated Hospital of Nanjing Medical University, Nanjing, China. Proximal gastrectomy (PG) was defined as resection of the proximal stomach and distal esophagus with esophagogastric anastomosis, and total gastrectomy (TG) was the removal of the entire stomach, proximal duodenum, and distal esophagus with esophagojejunal reconstruction. PG and TG were performed either via a transhiatal approach (transabdominal approach) or via a left thoracoabdominal incision (thoracoabdominal approach). Esophagogastrectomy was defined as resection of the proximal stomach and thoracic distal esophagus via a left thoracotomy (transthoracic esophagogastrectomy) with esophagogastric anastomosis in the chest. Neither radical transthoracic *en bloc* esophagectomy with resection of the proximal stomach (extended esophagectomy) nor Ivor-Lewis esophagectomy was performed in this study. The choice of operation type was based on the tumor site and surgeon preference with the aim of removing the entire primary tumor and its draining lymphatics. In all patients, D2 lymphadenectomy was performed and a macroscopically tumor-free proximal resection margin was achieved. Intraoperative frozen section margin evaluation was not routinely carried out in our institute.

### Pathologic Analysis

Tumor grade and stage were assessed by experienced gastrointestinal pathologists and classified according to the seventh edition of the TNM staging system of the American Joint Committee on Cancer for both gastric and esophageal diseases. All proximal margins were synchronously re-evaluated by two pathologists, and a positive proximal margin was defined as microscopic invasion of tumor cells at the esophageal transaction margin submitted *en face* on final paraffin sections.

### Statistical Analysis

Data are presented as frequencies, means (±SD), or medians with range. Statistical analyses were carried out by SPSS for Windows, version 13.0 (Statistical Package for the Social Sciences, SPSS, Inc., Chicago, IL). Univariate analysis was performed using a two-tailed χ^2^ test, unpaired Student’s t-test, or Wilcoxon test. Multivariate logistic regression analysis was used to identify risk factors that were independently associated with a positive proximal margin. A *P* value of <0.05 was considered statistically significant.

## Results

### General Information

A total of 483 patients with AGE who underwent complete gross resection were included in the present study. The mean patient age was 64 years (range, 32–85 years). There were 394 men and 89 women in this study, and the male:female ratio was 4.43∶1. The clinicopathologic characteristics of the patients are displayed in Table 1. Intraoperatively, the macroscopic length of the proximal tumor margin was more than 5 cm in most patients with AGE, and the average node harvest was 14.66±5.69. Most patients (n = 465) had type II and III tumors according to Siewert’s classification, and only 18 patients had type I tumors. For type I tumors, transthoracic esophagogastrectomy was performed. For type II and III tumors, 324 patients underwent PG and TG via a transhiatal approach, 139 patients underwent transthoracic esophagogastrectomy, and only 2 patients underwent TG via a thoracoabdominal approach. Twenty-nine transhiatal approaches were performed via laparoscopy.

**Table 1:** Comparison of demographic and pathologic factors between patients with positive and negative proximal margins.

Variables	Positive margin(n = 115)	Negative margin(n = 368)	*P* value
Age (years)	64.0±8.8	63.9±9.1	0.862
Gender			0.273
Male	98	296	
Female	17	72	
Average tumor size (cm)	4.2±1.5	4.0±2.0	0.324
Siewert type			0.003
I	10	8	
II & III	105	360	
Differentiation			0.905
Well-differentiation	31	103	
Poor-differentiation	84	265	
Gross type			0.774
Borrmann I	5	15	
Borrmann II III	105	299	
Borrmann IV	3	5	
Depth of tumor invasion			<0.001
T1	2(3.92%)	49	
T2	3(6.52%)	43	
T3	9(56.3%	7	
T4	101(27.3%)	269	
Node harvest	14.7±5.6	14.6±5.7	0.877
Node involvement			0.001
N0	27	140	
N1	22	82	
N2	36	81	
N3	30	65	
Stage			<0.001
p I	4	79	
p II	24	76	
p III	81	206	
p IV	6	7	
Vascular or lymphatic invasion			0.001
positive	41	72	
negative	74	296	
Perineural invasion			0.006
positive	47	101	
negative	68	267	

### Incidence of Positive Proximal Resection Margins

Among these 483 patients with AGE, there was no macroscopic cancer infiltration in the esophageal resection margin. However, 115 of the 483 patients (23.81%) had microscopically positive proximal margins. In this study, positive proximal margins were divided into mucosal infiltration and submucosal infiltration. Infiltration beyond the submucosal layer, including the muscle and even the outer fibrous layer, was grouped into submucosal infiltration. As shown in Table 2, both the mucosal and submucosal layers were infiltrated with cancer cells in 47 patients, and 3 patients had only positive mucosal margins. Positive mucosal margins were markedly associated with submucosal infiltration (*P*<0.001). However, 65 patients had positive submucosal margins without mucosal infiltration.

**Table 2:** Comparison of the positive proximal mucosal margin between positive proximal submucosal group and negative proximal submucosal group.

	Positive mucosal	Negative mucosal	χ^2^	*P* value
Positive sub-mucosal	47	65	159.015	<0.001
Negative sub-mucosal	3	368		

### Risk Factors Associated with Positive Proximal Margin in AGE

Risk factors for positive proximal margins in AGE by univariate and multivariate analyses are shown in Tables 1 and 3, respectively. On univariate analysis (Table 1), Siewert type, depth of tumor invasion, lymph node involvement, presence of vascular or lymphatic invasion, and presence of perineural invasion were significantly associated with positive proximal margins (*P*<0.05). These results indicate that positive proximal margins were strongly correlated with advanced-stage disease (*P*<0.001). The other factors, including age, gender, tumor size, differentiation, and Borrmann’s type, were not correlated with positive proximal margins in AGE.

**Table 3:** Multivariate logistic regression analysis of risk factors for a positive proximal margin.

Variable	Regression coefficient	Standard error	Odds ratio	95% CI for odds ratio	*P* value
Vascular or lymphatic invasion	0.557	0.244	1.745	1.082–2.851	0.022
Stage pIV	2.508	0.773	12.286	2.701–55.893	0.001

On multivariate analysis, the presence of vascular or lymphatic invasion and advanced-stage disease were independent risk factors for positive proximal margins in AGE (Table 3).

### Effects of Surgical Approach on Proximal Margin Status

The patients in this study underwent surgical treatment mainly via a transabdominal approach (n = 324) or transthoracic approach (n = 139), and only two patients underwent thoracoabdominal TG. As shown in Table 4, all 18 patients with type I tumors were excluded, and the effects of the surgical approach on the proximal margin status in patients with type II and III tumors were analyzed. The transthoracic approach group revealed a significantly higher incidence of positive proximal margins than the transabdominal approach group (*P = *0.022).

**Table 4:** Incidence of positive proximal margin between transthoracic group and transabdominal group in terms of Type II and III tumors.

	Positive proximal margin	Negative proximal margin	χ^2^	*P* valve
Transthoracic group	40	99	5.264	0.022
Transabdominal group	62	262		

Among 324 patients who underwent a transabdominal approach, 29 underwent laparoscopic procedures and 295 underwent open procedures. There was no difference in the incidence of positive proximal margins between these two groups (Table 5).

**Table 5:** Incidence of positive proximal margin between laparoscopic group and open group among patients undergoing transabdominal procedures in terms of Type II and III tumors.

	Positive proximal margin	Negative proximal margin	χ^2^	*P* value
Laparoscopic group	6	23	0.50	0.824
Open group	56	239		

## Discussion

The incidence of AGE has been increasing in Western countries [3]–[6], [10]. However, whether similar changes are occurring in Asia remains controversial. Kusano et al. [11] reported an increasing trend in the incidence of AGE from 1962 to 2005 in a large center in Japan, while Chung et al. [12] demonstrated that the ratio of patients with AGE to that of patients with non-AGE had not increased from 1992 to 2006 in a single institution in Korea. All of these previous research findings consistently indicated that type I tumors remain rare. Our retrospective study of the past 11 years showed a slight increase in the prevalence of AGE in our hospital (data not shown), which is consistent with previous observations [13], [14].

Radical surgical resection offers the best chance for curative treatment; however, residual cancer at the proximal resection margin poses a management dilemma for surgeons. The incidence of microscopically positive proximal resection margins reportedly varies from 2.5% to 58% among patients with AGE undergoing surgery with curative intent [4], [5], [7], [9]. It was an unfavorable 23.81% in our present series. The main risk factor for positive proximal margins of AGE was the depth of tumor invasion, as shown in our present study and other research [8], [9], [14]. We believe that the main reason for such a relatively high incidence of positive proximal margins in this series is that compared with other series, many more patients in our study (79.91%, 386/483) had advanced disease (T3 and T4 tumors) at the time of presentation. In Ito’s study, for example, only 56.63% (286/505) of all patients with AGE had T3 and T4 tumors, among which the incidence of positive proximal margins was 3% [5]. In addition, frozen section is not routinely used to assess the resection margin in our clinical practice due to its high false-negative rates (9–21%) [5].

It has been well documented that proximal margin invasion by AGE portends poor survival [4]–[6], [8]. However, a positive proximal margin has no significant effect on survival in patients with stage III and IV gastric cancer [8], and this group of patients usually succumbs to metastatic disease before the onset of anastomotic recurrence.

The risk factors for a positive proximal margin identified in this study included Siewert type, depth of tumor invasion, lymph node involvement, presence of vascular or lymphatic invasion, and presence of perineural invasion. Our observations confirmed that a positive proximal margin was strongly correlated with advanced-stage disease, similar to the findings of other reports [8], [9]. Specifically, vascular or lymphatic invasion and perineural invasion were associated with positive margins in this series. The presence of vascular or lymphatic invasion and perineural invasion may thus be forms of submucosal infiltration. Furthermore, multivariate analysis showed that the presence of vascular or lymphatic invasion was an independent risk factor for positive proximal margins in AGE.

Direct submucosal tumor extension or intramural spread is an important route of tumor spread [15]. Although a positive mucosal margin was markedly associated with submucosal infiltration, 56.52% (65/115) of patients had positive submucosal margins without mucosal infiltration in the present study. This phenomenon should attract the attention of surgeons. Failure to appreciate this fact will lead to transection of the tumor at the esophageal margin [9], which may explain the relatively high prevalence of overall positive margins in the present study. The degree of intramural extension by AGE is strongly correlated with T stage [5]. Our data are consistent with this observation. The incidence of residual cancer at the resection margin was 5.15% in T1 and T2 tumors, while it was 28.5% in T3 and T4 tumors (*P*<0.05).

Achieving clear resection margins for AGE can be challenging given its propensity for submucosal spread [5]. There was no agreement on the measurement of margin lengths in this study. Generally, the surgeons judged the adequacy of resection margin length by palpation and gross inspection, and the *in situ* length present intraoperatively before completion of the resection was more than 5 cm in most patients with AGE; however, this was unreliable due to submucosal infiltration. Because of the phenomenon of specimen shrinkage, these lengths measured postoperatively do not necessarily reflect corresponding *in situ* lengths intraoperatively before completion of the resection. Siu et al. [16] reported that proximal margin lengths measured on prefixed esophageal resection specimens were only 44% of the corresponding lengths measured *in situ* before completion of resection.

Extended resections, including D2 lymphadenectomy, have been proposed in an attempt to reduce the incidence of positive margins and improve locoregional control and survival [4]. The surgical approach to AGE is not standardized and varies with the experience of the surgeons as well as the pathological features of these lesions [17]. Two surgical phase III trials have indicated that type I AGE should be treated surgically as esophageal cancer, while types II and III should be regarded as true gastric cancer [6]. Most series do not demonstrate a survival benefit for one operative approach over another [4]. The achievement of clear proximal margins should be the common objective for all approaches. The operative approach may be individualized to meet this goal [4], [5], which depends on the overall condition of patient, Siewert type, T stage, and N stage. Generally, extended esophagectomy is recommended for type I tumors, as for esophageal cancer [4], [10]. No patients underwent extended esophagectomy in this study, which may also be ascribed to the high incidence of positive margins. For type II and III tumors, preoperative assessment is of great importance [10]. The present study indicates that the depth of tumor invasion is one of the risk factors for positive proximal margins. Preoperative T staging can be accurately achieved by modalities such as endoscopic ultrasound, and this may permit a tailored approach to the extent of esophageal resection for patients with AGE [4]. A proximal gross margin length of at least 6 cm is required to achieve a microscopically negative proximal margin for T3 and T4 cancers [5]. Barbour et al. indicated that grossly negative *ex vivo* esophageal margins of more than 3.8 cm were associated with a favorable outcome for patients with Siewert type II and III tumors following radical resection with removal of more than 15 lymph nodes. Further analysis revealed that the association between improved outcomes and extended esophageal margins was confined to those patients with greater than T1 tumors and fewer than seven positive lymph nodes [4]. Large prospective series have not shown superior oncologic outcomes for TG compared with PG for proximal gastric and GEJ cancer, nor has esophagectomy been found to improve outcomes over extended gastrectomy for GEJ cancer [4]. A randomized trial also failed to demonstrate a clear benefit for extended transthoracic esophagectomy over transhiatal esophagectomy [17]. In the present study, transthoracic gastrectomy with distal esophagectomy revealed a significantly high incidence of positive proximal margins compared with the transabdominal approach, indicating that transthoracic gastrectomy with distal esophagectomy is not suitable for type II and III tumors. However, this finding should be interpreted with caution. Abdominal transhiatal resection may be performed for localized, noninfiltrating tumors and esophageal involvement of less than 2 cm. However, infiltrating, poorly differentiated, or Borrmann III–IV tumors require a thoracoabdominal extended gastrectomy to achieve a longer margin of clearance [12]. When esophageal involvement is more than 3 cm, no surgical procedure is curative, and the literature demonstrates that extended aggressive surgery has no benefits [15]. Despite little evidence, we propose a thoracoabdominal approach (extended gastrectomy via a left thoracoabdominal incision) or Ivor-Lewis esophagectomy for selected patients with AGE, especially those with advanced type II and III tumors, to reduce the incidence of a positive proximal margin.

The advantage of laparoscopic resection for gastric cancer is well recognized [10], [18]. In our hospital, a laparoscopic approach is routinely undertaken for patients with gastric cancer. This observation of similar positive margin confirmed the safety and feasibility of this operation.

This study had several limitations. The true margin length in each case was not accurately recorded in this retrospective study, and it was very difficult to ascertain type II from type III tumors. Our recommendation on the surgical approach to AGE mainly depended on our findings and the literature review. Because of the small sample size and single-institution experience, larger prospective studies are required to validate our findings in the Chinese patient population. Because the cases were relatively new, there were no survival data in this study. We will continue to follow these patients and determine the effects of positive proximal margins on survival in this series. In addition, the present study did not evaluate the effects of neoadjuvant therapy on the proximal margin status following resection of AGE. Neoadjuvant chemoradiotherapy was considered to reduce the stage, enhance the resectability, and improve the overall survival of patients with gastric cancer [19]. However, whether neoadjuvant therapy reduces the incidence of positive proximal margins remains unclear. Neoadjuvant therapy has been conducted in our hospital for advanced gastric cancer, and its effect on the proximal margin status of AGE will be investigated in the future. Furthermore, endoscopic ultrasound and frozen section will be adopted routinely in our practice to reduce the incidence of positive proximal margins in AGE from now on.

In conclusion, residual cancer at the proximal resection margin remains a major issue for the surgical treatment of AGE in China. To obtain clear margins, an individualized surgical approach depending on the preoperative evaluation and intraoperative findings should be undertaken.
